# Precision Cutting of CF/PEEK by UV Nanosecond Laser for On-Orbit Manufacturing Applications

**DOI:** 10.3390/mi17010093

**Published:** 2026-01-11

**Authors:** Wenqiang Wu, Bing Wei, Yu Huang, Congyi Wu

**Affiliations:** 1 School of Mechanical Engineering, Hubei University of Technology, Wuhan 430068, China; wuwenqiang1231@outlook.com; 2 School of Mechanical Science and Engineering, Huazhong University of Science and Technology, Wuhan 430074, China; yuhuang_hust@hust.edu.cn (Y.H.); wucongyi@hust.edu.cn (C.W.)

**Keywords:** CF/PEEK, on-orbit cutting, UV nanosecond laser, by-product

## Abstract

On-orbit cutting is a critical process for the on-orbit manufacturing of carbon fiber reinforced polyetheretherketone composites (CF/PEEK) truss structures, with pulsed laser cutting serving as one of the feasible methods. Achieving high-quality cutting of CF/PEEK remains a major challenge for on-orbit manufacturing. Therefore, the cutting process of CF/PEEK prepreg tape was studied by an ultraviolet (UV) nanosecond laser. A three-factor, five-level orthogonal experiment was carried out to analyze the influence of laser repetition rate (LRR), laser cutting speed (LCS), and laser scanning times (LCTs) on cutting quality. The ablation mechanism dominated by the photothermal effect between the UV nanosecond laser and CF/PEEK was analyzed, and the by-products in the cutting process were explored. Finally, the optimal cutting quality (the width of slit (W_s_) = 41.69 ± 3.54 μm, the heat-affected zone (HAZ) = 87.27 ± 7.30 μm) was obtained under the process conditions of LRR 50 kHz-LCS 50 mm/s-LCT 16 times. The findings show that the W_S_ and HAZ increase with the increase in LRR and LCT and the decrease in LCS, and the carbon fiber decomposes and escapes due to the photothermal effect.

## 1. Introduction

The demand for large-scale space infrastructure such as deep space probes and communication antennas has promoted the development of on-orbit manufacturing technology for trusses [1,2,3,4]. CF/PEEK possesses excellent mechanical properties [5], heat resistance, radiation resistance [5,6], toughness, and fatigue stability [7,8,9], making it a preferred material for on-orbit truss manufacturing and widely used in the aerospace sector for fabricating various components [10,11]. Yuanhao Xia et al. [12] reported a method for producing high-performance CF/PEEK thin-walled tubes using a traction winding technique, which overcomes the limitations of core mold size while enabling the continuous production of tubes with ø20 mm. Jiayong Yan et al. [13] described a process for rod pultrusion–winding based on CF/PEEK prepreg tape, achieving the rapid and efficient fabrication of slender, continuous, high-stiffness triangular trusses with low energy consumption. To meet the construction requirements of diverse space structures, a viable approach involves cutting structural members first and then assembling them into various space structures using dedicated connectors. Furthermore, the effective cutting of continuous CF/PEEK trusses is also necessary.

At present, the cutting of carbon fiber mainly relies on traditional mechanical cutting [14,15,16]. Traditional mechanical milling and sawing offer high efficiency, which is suitable for mass production in factories or rapid removal of a large number of materials. However, the high degree of complexity inherent in process equipment presents a significant challenge for fulfilling the lightweight design prerequisites essential for on-orbit manufacturing. In recent years, alternative traditional machining processes—including ultrasonic machining, abrasive water jet machining, electrical discharge machining, and laser-based methods—have also seen widespread adoption. Ultrasonic machining [17] combines ultrasonic vibration machining technology with traditional mechanical milling, which significantly enhances material removal rates and improves machining precision and surface quality, while simultaneously reducing cutting forces, thermal impact, and tool wear—but at the cost of increased complexity in process system design. Abrasive water jet machining [18,19] is a typical cold processing method. It uses fine particles of abrasives such as garnet to cut materials under the drive of water flow, avoiding problems such as material thermal degradation and matrix melting. However, the need for cutting water and wastewater treatment pose challenges to on-orbit manufacturing. Electrical discharge machining [20] is based on the phenomenon of electrical corrosion during pulsed spark discharge between the tool and the workpiece. The workpiece has a high surface finish, but there is electrode wear during long-term use, thus requiring regular electrode replacement. In order to meet the on-orbit cutting requirements of CF/PEEK truss structures, it is urgent to find a cutting method with high cutting accuracy, simple composition, and near-zero by-products.

Laser cutting [21,22] offers advantages, including non-contact processing, high cutting precision, simple system composition, and strong material adaptability, making it one of the feasible methods for on-orbit cutting of CF/PEEK. Continuous lasers provide high power, large spot diameters, and high processing efficiency, but cause severe thermal ablation. Ultra-short pulse (ps, fs) lasers deliver large single-pulse energy, small spot diameters, and minimal thermal impact; however, they are limited by laser power and low cutting efficiency. Compared to continuous and ultra-short pulse lasers, nanosecond lasers offer higher power and smaller spot diameters, making them a preferred solution for CF/PEEK cutting [23]. UV laser (355 nm), compared to infrared (1064 nm) and green light (532 nm), possesses higher photon energy and smaller spot diameters, enabling smaller HAZ and better machining accuracy, thus representing the optimal solution for CF/PEEK cutting [24,25]. However, the carbon fiber evaporation temperature exceeds 3000 °C, while the PEEK melting point is only 343 °C [26]. This significant difference in thermal properties results in a substantial HAZ. On-orbit laser processing technology still faces several technical bottlenecks, the mechanisms underlying the cutting process and the physicochemical properties of the resulting by-products lacking systematic analysis. Therefore, cutting parameters and damage areas still require optimization, and the interaction mechanism between UV nanosecond lasers and CF/PEEK needs systematic investigation.

In this study, the cutting process of CF/PEEK prepreg tape was investigated using a UV nanosecond laser processing platform. Firstly, an evaluation system for cutting quality was established, utilizing the Ws and HAZ as key metrics. Secondly, the optimal cutting quality (W_S_ = 41.69 ± 3.54 μm, HAZ = 87.27 ± 7.30 μm) was obtained by a three-factor and five-level orthogonal experiment under the optimal process parameters, and a systematic analysis was conducted to evaluate the impact of process parameters on cutting quality. Finally, the microstructure and solid by-products were characterized by scanning electron microscope (SEM) and Raman spectroscopy. The cutting process and gas by-products were analyzed by thermogravimetric–infrared spectroscopy (TG-IR) and pyrolysis–gas chromatography–mass spectrometry (Py-GC/MS), which provided theoretical support for the by-product treatment of the CF/PEEK on-orbit ultraviolet nanosecond laser cutting system. However, the influence mechanisms of typical space environmental factors, such as vacuum, extreme temperatures, and microgravity, on material behavior, thermal management, and debris dispersion characteristics during the cutting process remain unclear and warrant in-depth investigation.

## 2. Experiment

### 2.1. Materials

The processing material was unidirectional carbon fiber prepreg tape, supplied by Jiangsu Junhua Special Polymer Materials Co., Ltd. (Changzhou, China), with specific parameters detailed in Table 1.

### 2.2. Laser Cutting System

**Figure 1:**
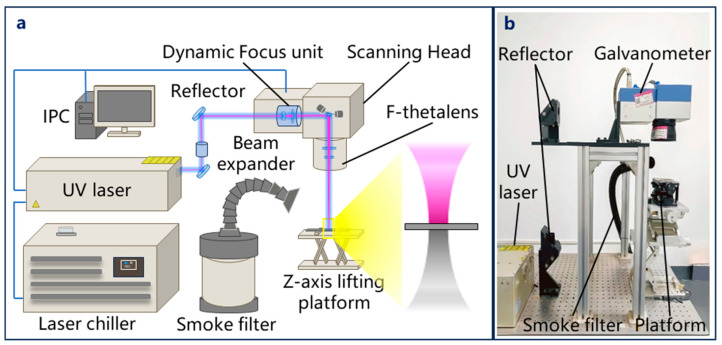
(**a**) Schematic diagram and (**b**) physical diagram of the UV nanosecond laser cutting system.

**Figure 2:**
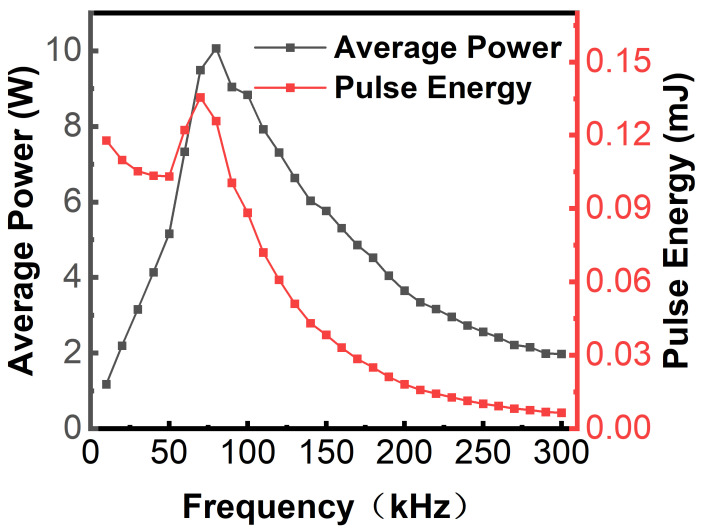
Variation in average laser power and pulse energy as a function of LRR.

As shown in Figure 1, the laser (Poplar-355-18A, Wuhan Huaray Precision Laser Co., Ltd., Wuhan, China) employed operates at a working wavelength of 355 nm, with the corresponding parameters provided in Table 2. The laser beam is directed through a reflector, while its diameter is increased by a beam expander. The optical path system is further equipped with a high-precision dynamic scanning galvanometer and a telecentric field lens (F-Theta Ronar lens, Linos, Göttingen, Germany) featuring a focal length of 167 mm. The high-precision dynamic scanning galvanometer contains a dynamic focusing unit (VarioScande 20i, Scanlab, Puchheim, Germany) and a two-dimensional scanning unit (IntelliSCAN 14, Scanlab, Puchheim, Germany). The laser is deflected by the galvanometer, and finally focused by the telecentric field lens to achieve spot movement on the material surface. The auxiliary system includes a water cooler and a smoke filter. An industrial personal computer (IPC) is used to control the laser and galvanometer. The average power (*P_avg_*) is adjusted by changing the *LRR*. *P_avg_* is measured using a power meter, and the single-pulse energy (*E_p_* = *P_avg_*/*LRR*) is determined by *P_avg_*, the corresponding relationship between *P_avg_*, *E_p_* and LRR is illustrated in Figure 2. The single-pulse fluence (*F*) is defined as

$$F=\frac{E_{p}}{A}=\frac{P_{avg}/LRR}{\pi {\left(\frac{d_{0}}{2}\right)}^{2}}$$

The peak fluence (*F_peak_*) is defined as

$$F_{peak}\approx \frac{F}{\tau }$$

Table 3 summarizes the calculated typical parameters derived from the measured average power.

### 2.3. Design of Experiment

Based on the extensive previous literature, the quality of material cutting is predominantly influenced by the LRR, LCS, and LCT. Pre-experimental data revealed significant slit quality variations within these operational windows: LRR 10–30 kHz, LCS 10–90 mm/s, and LCT 8–24 times. Therefore, in order to systematically evaluate parameter effects on cutting quality, a three-factor, five-level orthogonal experiment was designed. Parameter levels are detailed in Table 4. The experiments were performed in air. The focused laser beam irradiated the material surface and scanned along a path perpendicular to the fiber direction, with an exhaust system operating to eliminate process fumes. To ensure data reliability, four repetitions were carried out for each processing parameter combination.

### 2.4. Characterization

The surface morphology, slit, and HAZ after laser cutting were photographed and measured using a digital microscope with ultra-depth of field (VHX-7000, KEYENCE, Osaka, Japan). Microstructural features were examined via scanning electron microscopy (FEI Sirion 200, FEI Company, Hillsboro, OR, USA). Solid by-product was performed by a Raman spectrometer (HR800, HORIBA Jobin Yvon, Longjumeau, France). Thermogravimetric–Fourier transform infrared spectroscopy (TGA 8000 + Frontier FTIR, PerkinElmer, Waltham, MA, USA) was used to monitored real-time mass loss and gaseous emissions. Rapid pyrolysis experiments in helium atmosphere simulated material ablation processes. The pyrolytic gas products were characterized both qualitatively and quantitatively using gas chromatography–mass spectrometry (Agilent 7890A, Agilent Technologies, Santa Clara, CA, USA).

## 3. Results and Discussion

### 3.1. Evaluation of Laser Cutting Seam

**Figure 3:**
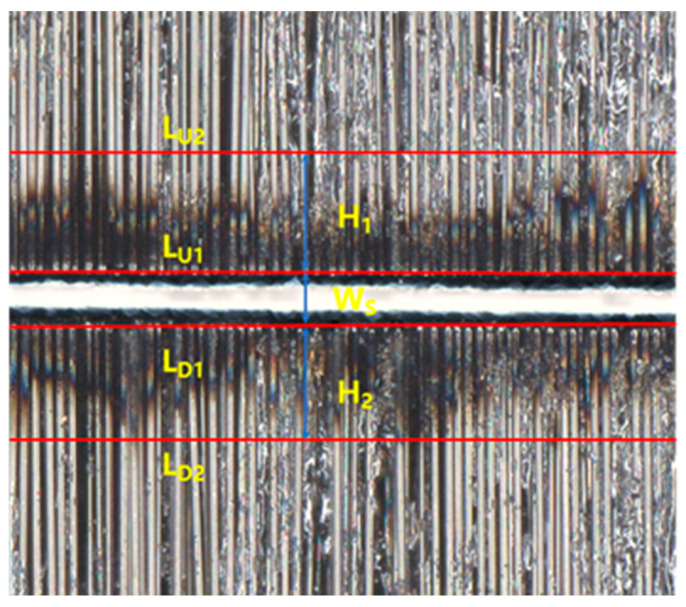
Schematic illustration of the evaluation methodology for laser cutting quality.

The surface topography of the cut workpiece was measured using a depth-of-field microscope, and a representative result is shown in Figure 3. The slit divided the carbon fiber prepreg into two parts, exposing carbon fibers along the kerf edges as distinctive bright white spots. Reference line L_U1_ is defined along the upper slit edge, and a parallel line L_D1_ of L_U1_ is constructed to maximize coincidence with lower-edge white spots. The distance between L_U1_ and L_D1_ is W_S_. The transition region between kerf and undamaged material constitutes the HAZ. The boundary of the HAZ often exhibits a non-linear morphology. The parallel line L_U2_ of L_U1_ coincides with the highest edge of the HAZ. The parallel line L_D2_ of L_D1_ coincides with the lowest edge of the HAZ. The distance between L_U1_ and L_U2_ is H_1_, and the distance between L_D1_ and L_D2_ is H_2_, then the HAZ = Max (H_1_, H_2_).

To achieve high-quality and efficient cutting, with “cut-through” as the core constraint, we prioritize identifying parameters that minimize the HAZ, and subsequently determine the optimal value for Ws from among those. This objective is based on their fundamental influence on the joint performance of the component: “cut-through” fulfills our functional requirement; controlling the HAZ aims to limit thermal damage to the material, thereby preserving the effective load-bearing cross-section at the joint; and minimizing Ws is intended to reduce material removal, ensuring the dimensional fit required for subsequent connections. Concurrently, cutting efficiency is also taken into consideration to achieve the optimal overall process performance.

**Figure 4:**
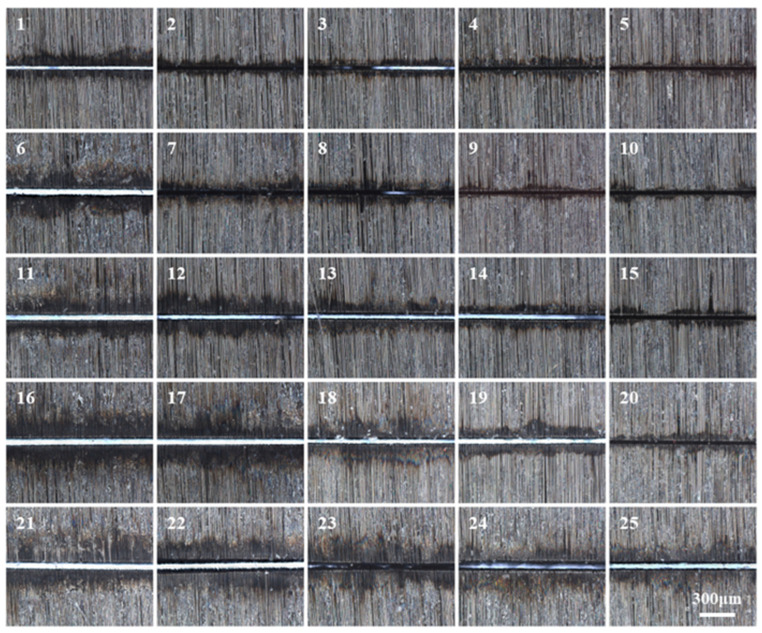
Representative microscopic images of the individual samples.

The average value of the repeated experimental results is recorded in Table 5, and the morphological characteristics after typical cutting are shown in Figure 4. Based on a comprehensive evaluation of the chart data, both Parameter Set 1 and Parameter Set 13 satisfy the prerequisite of “cutting through” and enable effective processing. However, the two sets exhibit distinct performance advantages in key metrics: Set 1 demonstrates superior performance in minimizing Ws, whereas Set 13 excels in the primary optimization objective—namely, minimizing HAZ—thereby providing more reliable assurance for the structural strength of the joint. From an engineering perspective, Parameter Set 13 also achieves a significant improvement in cutting efficiency, with its LCS increased by a factor of 2.5, which holds considerable value for reducing the actual production cycle time.

### 3.2. Orthogonal Experiment Analysis of Variance

Analysis of variance (ANOVA) [27,28] assesses the contribution of controllable factors to experimental outcomes by decomposing the total variation, and subsequently determines their statistical significance. According to the differences in the sources of variation, ANOVA can be further distinguished between the main effect analysis (testing the independent influence of a single factor) and the interaction effect analysis (testing the combined effect of multiple factors). For variability assessment, the range (R) is calculated as the difference between the maximum and minimum values within the sample, which is used to characterize the discrete range of the data. Based on the orthogonal experimental design, the optimal level of each factor can be efficiently identified, and the global optimal parameter combination can be determined by comparing the marginal effects of the level combination.

**Figure 5:**
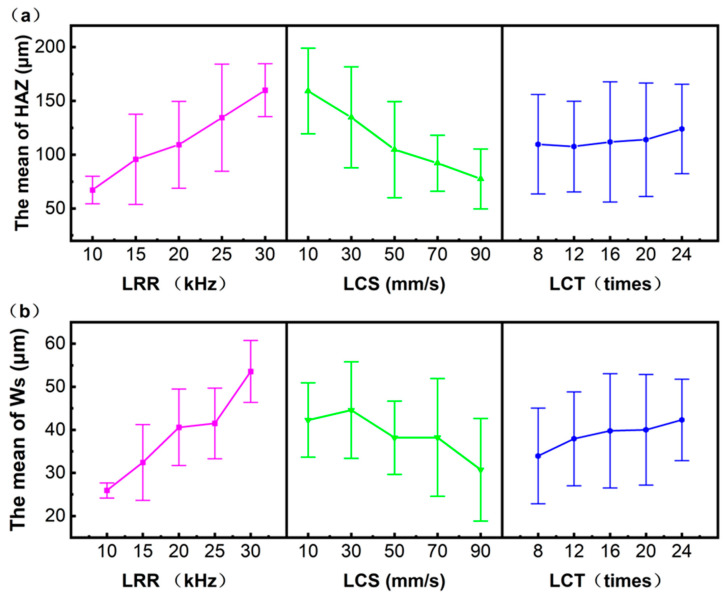
Impact assessment of different process factors based on (**a**) HAZ and (**b**) W_s_.

When the HAZ is used as the evaluation index, the results of variance analysis are as shown in Table 6, and the results of range analysis are as shown in Figure 5a. The results of variance analysis of HAZ show that LRR, LCS, LCT, and the interaction LRR*LCS had significant effects on the HAZ (*p* < 0.001). The range analysis results of HAZ show that, for the LRR, the HAZ is positively correlated with the LRR. At 10 kHz, the minimum HAZ is 67.31 μm. At 30 kHz, the maximum HAZ reaches 160.02 μm (R = 92.71 μm), with optimal Level 1. For the LCS, HAZ is negatively correlated with the LCS, the range is 81.76 μm, and the optimal level is Level 5. For the LCT, when LCT increase from 8 to 16, HAZ decreases from 109.82 μm to 107.70 μm. When LCT increases from 16 to 24, HAZ rises to 124.07 μm, the range is 16.37 μm, and the optimal level is Level 2. Thus, the optimal parameter combination within the experimental orthogonal range is 1 (10 kHz)—5 (90 mm/s)—2 (12 scans). However, as demonstrated by the Group 5 results (parameters: 1 (10 kHz)—5 (90 mm/s)—4 (20 scans)), the carbon fiber prepreg tape remains uncut. Therefore, Combination 1-5-2 also fails to achieve complete cutting. In addition, because R1 > R2 > R3, LRR exerts the strongest influence on HAZ width, while LCT has the weakest.

When the W_S_ is used as the evaluation index, the outcomes of the variance analysis are as presented in Table 7, and the findings from the range analysis are displayed in Figure 5b. The results of variance analysis of W_S_ shows that LRR, LCS, LCT, and the interaction LRR*LCS have significant effects on W_S_ (*p* < 0.001). The range analysis results of W_S_ show that, as the LRR increases from 10 kHz to 30 kHz, the average output power increases from 1.18 W to 3.16 W, while E_p_ slightly decreases from 0.118 mJ to 0.105 mJ, and WS increases from 25.956 μm to 53.558 μm. The range of WS is 27.603 μm, and the optimal level is Level 1. For the LCS, W_S_ is wider at 10 mm/s and 30 mm/s. When the LCS increases to 50 mm/s and 70 mm/s, the W_S_ decreases. At 90 mm/s, W_S_ is minimized (30.748 μm). The range is 13.851 μm, and the optimal parameter level is Level 5. For the LCT, as the LCT increases, the W_S_ increases slightly, the range is 8.388 μm, and the optimal parameter level is Level 1. Therefore, when the process combination is 1 (10 kHz)—5 (90 mm/s)—1 (8 times), the minimum W_S_ can be obtained. However, as shown in the 20th group of processing results, the Parameter Set 4 (25 kHz)—5 (90 mm/s)—1 (8 times) fails to cut through the carbon fiber prepreg tape. Consequently, Combination 1 (10 kHz)—5 (90 mm/s)—1 (8 times) also fails to cut through the CF/PEEK prepreg tape. In addition, since R1 > R2 > R3, for the W_S_, LRR has the strongest influence on W_S_, while LCT has the weakest.

The error bar observed in the results presented in Figure 5 can be primarily attributed to three inherent sources of microstructural variability in the composite material. The stochasticity of fiber distribution, gradients in resin impregnation, and fluctuations in interfacial bonding collectively introduce spatial uncertainties in laser energy deposition and heat conduction, which are macroscopically manifested as a significant measurement error.

The response mechanism of cutting quality (HAZ and W_s_) to process parameters can be attributed to the direct manifestation of the thermal accumulation effect of laser energy. At a fixed LCS, when the LRR increases from 10 kHz to 30 kHz, the E_p_ slightly decreases from 0.118 mJ to 0.105 mJ, but the P_avg_ increases significantly. This results in a high temporal overlap between adjacent pulses, where the residual heat generated by the previous pulse has not yet dissipated before the subsequent pulse energy is continuously delivered, leading to significant thermal accumulation in the time domain. This effect can be quantified by the pulse overlap rate (Overlap = (1 − (LCS/(LRR × d_0_))) × 100%). A higher overlap rate corresponds to more pronounced thermal accumulation, causing HAZ and W_s_ to expand. At a fixed LRR, reducing the LCS prolongs the laser’s dwell time in the same area, significantly increasing the energy deposited per unit length (E_L_ = (P_avg_/LCS) × LCT). Meanwhile, although the laser energy density depends on the single-pulse energy, at low scanning speeds, multiple high-energy-density pulses overlap spatially to a great extent, further intensifying energy concentration and thermal diffusion in the spatial domain. The synergistic effect of these factors causes heat to diffuse both deeper into and laterally across the material, leading to an expansion of the thermal damage range (HAZ) in the fibers and matrix, as well as an increase in material removal (larger W_s_).

**Figure 6:**
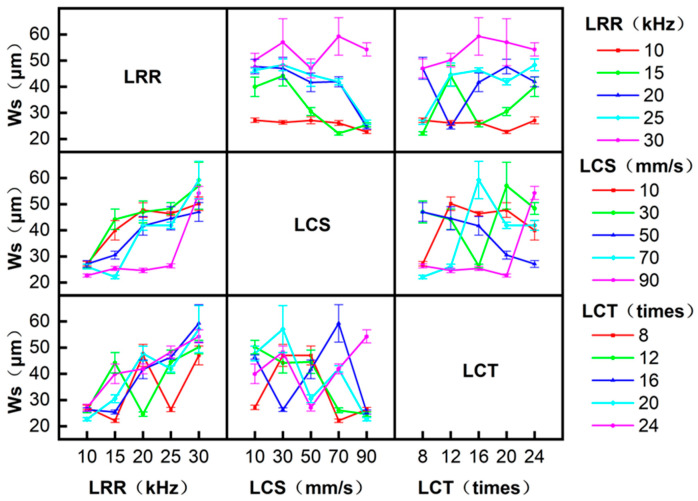
Interactions among processing parameters influencing the straightness of the W_S_.

**Figure 7:**
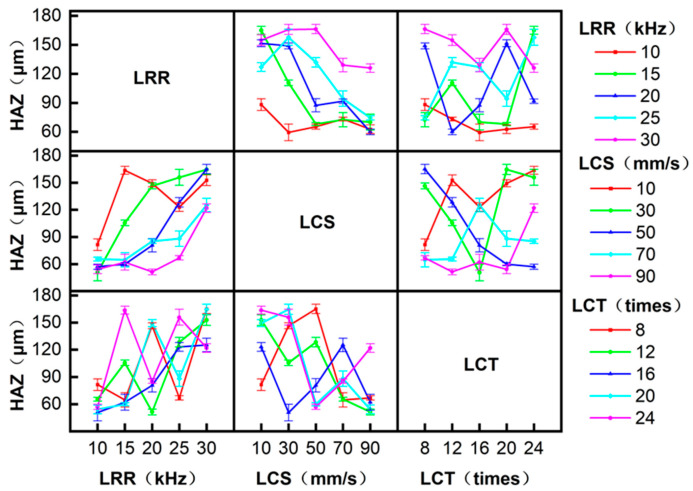
Interactions among processing parameters influencing the straightness of the HAZ.

Interaction analysis in ANOVA examines correlations between factors. Figure 6 and Figure 7 show the effects of the interaction between LRR, LCS, and LCT on the W_S_ and HAZ, respectively. For the W_S_, with the increase in LRR, the W_S_ increases gradually under different LCS conditions. At LRR = 10 kHz and 30 kHz, LCS shows negligible influence on W_S_. At LRR = 15, 20, and 25 kHz, LCS significantly affects W_S_, with W_S_ decreasing as LCS increases. The interaction of other parameters has no obvious effect on the W_S_. For the HAZ, with the increase in LRR, the HAZ gradually increases under different LCS conditions. When LRR is 10 and 30 kHz, LCS has little effect on the HAZ. At LRR levels of 15, 20, and 25 kHz, LCS has a great influence on the HAZ, and the HAZ decreases with the increase in LCS. The interaction of other parameters has no obvious effect on the HAZ.

### 3.3. Mechanism of the Nanosecond UV Laser Cutting

**Figure 8:**
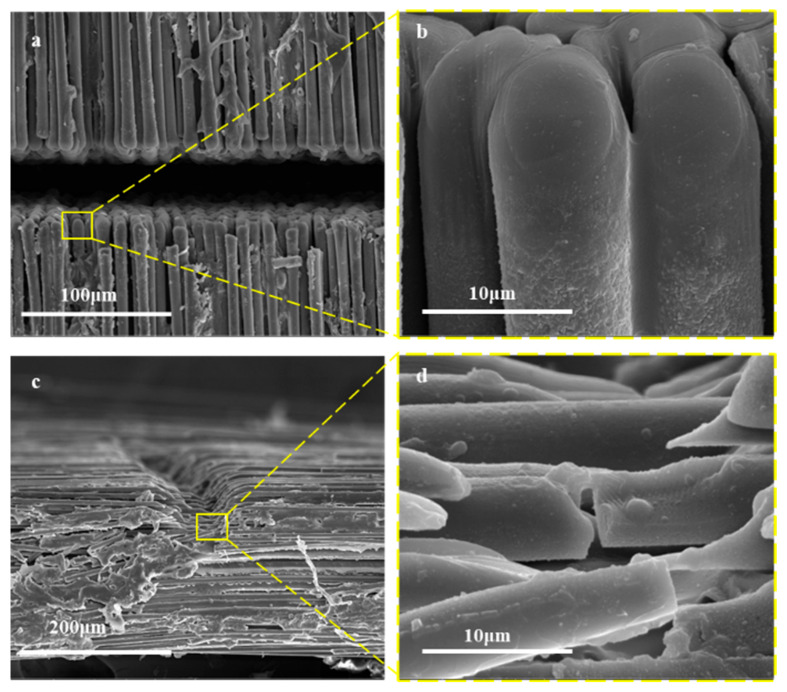
SEM images of the (**a**) slit surface and (**b**) partial enlarged view; SEM images of the (**c**) slit section and (**d**) partial enlarged view.

To investigate how process parameters affect machining quality, a fundamental understanding of the interaction mechanism between the UV nanosecond laser and CF/PEEK composites is essential. Firstly, the post-cutting microstructure was characterized using SEM. Figure 8 shows the surface view of the slit, the cross-section view of the uncut sample and its local enlarged view. As shown in Figure 8a, the section of the slit is neat, and there are a small number of fiber breakage gaps and broken fiber residues on both sides of the slit. A small amount of matrix adheres to partial fibers, featuring smooth edges—indicating that the matrix is vaporized to form a HAZ due to the photothermal effect or fiber thermal conductivity during the ablation process. As shown in Figure 8b, the fiber section has a certain taper, and the incision is smoother than the original material. This indicates that the carbon fibers were vaporized and decomposed due to the photothermal effect. Figure 8c shows the processing cross-section under the condition of 20 kHz—50 mm/s—four times. The incision presents a U-shape, and the matrix at the edge of the slit disappears, exposing the fiber. Figure 8d is a local enlarged image at the bottom of the slit. Some of the upper surfaces of the fibers have depressions with clean fracture planes, suggesting thermal stress-induced failure.

**Figure 9:**
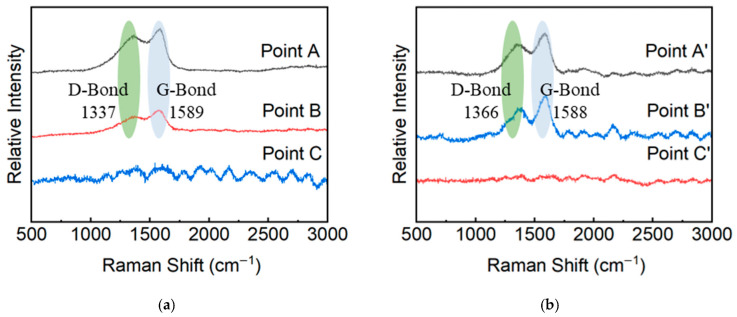
Raman measurements of the cutting seam (**a**) before and (**b**) after cleaning.

To investigate the cutting mechanism of the ultraviolet nanosecond laser, the sample was placed in an ultrasonic cleaner containing an ethanol solution (95% concentration) and cleaned for 10 min to remove residual processing dust and debris from the surface. The solid-phase composition of the post-cut material surface was examined using Raman spectroscopy [29,30]. A 532 nm excitation source was used to analyze three regions—kerf edge (Point A), HAZ (Point B), and undamaged zone (Point C), with spectra compared before and after cleaning. As shown in Figure 9a,b, Point A and Point B have obvious carbon characteristic peaks of D band (1350 cm^−1^) and G band (1580 cm^−1^), and there are no obvious other characteristic peaks. This indicates complete matrix removal at the kerf edge and HAZ, exposing carbon fibers without chemical alteration or contamination. After cleaning, the I_D_/I_G_ ratio at point A decreased from 0.966 to 0.943, while at point B it decreased from 0.947 to 0.908. This indicates that the cleaning process removed a small amount of disordered carbon layers left by processing, thereby enhancing the structural order of the surface, while the surface morphology remained essentially unchanged.

**Figure 10:**
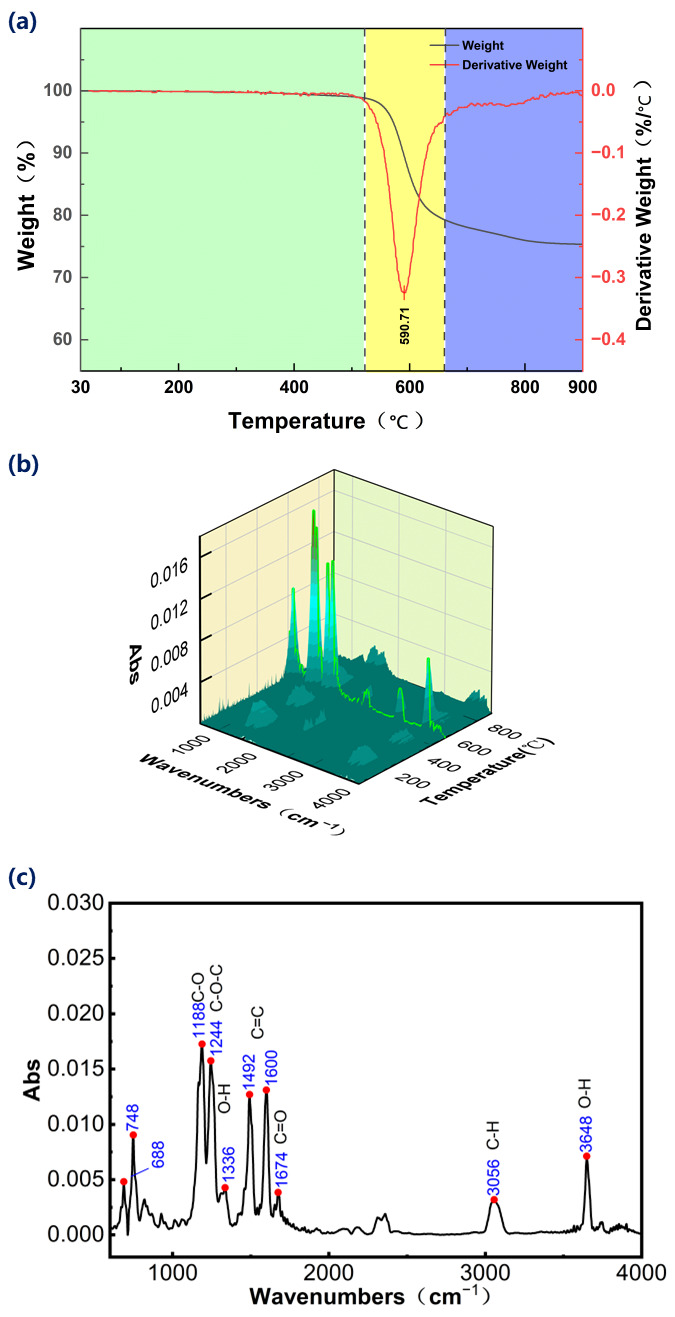
(**a**) TG and DTG curves of the CF/PEEK, (**b**) infrared spectral analysis of gaseous products released during the thermal degradation of CF/PEEK, (**c**) corresponding spectral profile obtained at 590 °C.

To further analyze the dynamic evolution process of carbon fiber and substrate during UV nanosecond laser cutting, TG-FTIR technology was employed in a helium atmosphere to characterize material decomposition kinetics and gaseous by-products. The melting point of PEEK is approximately 343 °C while carbon fibers vaporize over 3000 °C in air [31]. Consequently, within the temperature range of the thermogravimetric test, only PEEK decomposition occurs, and the carbon fiber remains almost entirely thermally stable. Furthermore, the PEEK does not fully decompose. As shown in Figure 10a, the main decomposition temperature of PEEK is 520–660 °C, and the maximum decomposition rate is reached at 589.58 °C. After the temperature reaches 650 °C, the matrix material still decomposes slowly.

Figure 10b shows the infrared spectrum of CF/PEEK gaseous decomposition products at temperatures from 30 °C to 900 °C. No obvious infrared absorption peak was observed below 500 °C. As the temperature increased, the weak peak gradually increased and reached a maximum at 590 °C. Figure 10c shows the infrared spectrum when the temperature reaches 590 °C. This stage is a more intense stage of polyetheretherketone decomposition. The absorption peak at 3648 cm^−1^ is related to the stretching vibration of the O-H bond, indicating that the gas product contains water vapor, which may be derived from the ether bond breaking and recombination reaction. The absorption peak at 1244 cm^−1^ represents the C-O-C asymmetric stretching vibration, indicating that there are still residual ether bonds. The aromatic peaks (3056, 1600, 1492) have significant intensity, and the representative products are polycyclic aromatic hydrocarbons such as benzene and naphthalene, which confirms that the PEEK aromatic ring remains stable at 590 °C, and the cracking is dominated by side chain breakage and recombination. The absorption peaks of 688 cm^−1^ and 748 cm^−1^ are aromatic compounds, which are the products of aromatic ring recombination. The characteristic peaks at 1188 cm^−1^ (C-O) and 1336 cm^−1^ (O-H) are phenols, which represent the products of phenol and cresol. The peak of quinone (1674 cm^−1^) is weak, indicating that the main decomposition path is non-oxidizing thermal cracking. In summary, the pyrolysis of PEEK at 590 °C is dominated by aromatic ring structure retention and ether bond cleavage to generate water, resulting in a large number of monocyclic/polycyclic aromatic hydrocarbon fragments, and limited oxidation side reactions.

**Figure 11:**
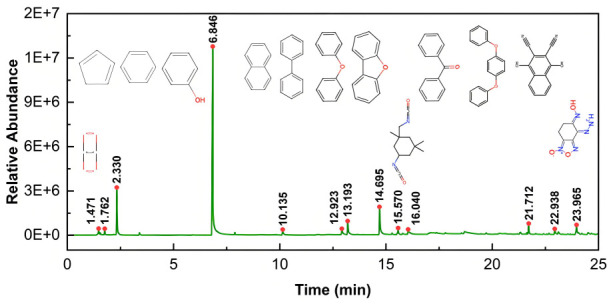
Ion current of evolved gases.

To identify the potential hazardous gas components that may be generated from the thermal decomposition of the PEEK, we conducted an analysis of CF/PEEK material using Py-GC/MS under a helium atmosphere. As show in Figure 11 and Table 8. The Py-GC/MS analysis results indicate that, under thermal influence, the PEEK matrix primarily undergoes reactions such as main chain scission, ether bond cleavage, and degradation of aromatic ring structures. The gaseous products generated include aromatic compounds such as phenol, benzene, biphenyl, diphenyl ether, and dibenzofuran, along with a small amount of carbon dioxide. Among these, phenol is one of the main decomposition products, reflecting the characteristic main chain scission of PEEK. Products such as biphenyl and diphenyl ether directly originate from the pyrolysis of PEEK structural units, while dibenzofuran is likely formed through the rearrangement of cleaved ether bonds. Since the experiment was conducted in an inert atmosphere, the influence of oxidation reactions is excluded. Therefore, the detected carbon dioxide mainly results from the cleavage of carbonyl groups within the material’s own structure. It is important to note that substances such as benzene, phenol, and naphthalene exhibit high toxicity and can irritate the respiratory tract, eyes, and skin. Long-term exposure may lead to cumulative health damage. Therefore, during actual laser processing operations, personal protection for personnel must be emphasized. It is recommended to wear safety goggles and respiratory protection masks, and operate in a well-ventilated environment to minimize the risk of exposure to harmful gases.

**Figure 12:**
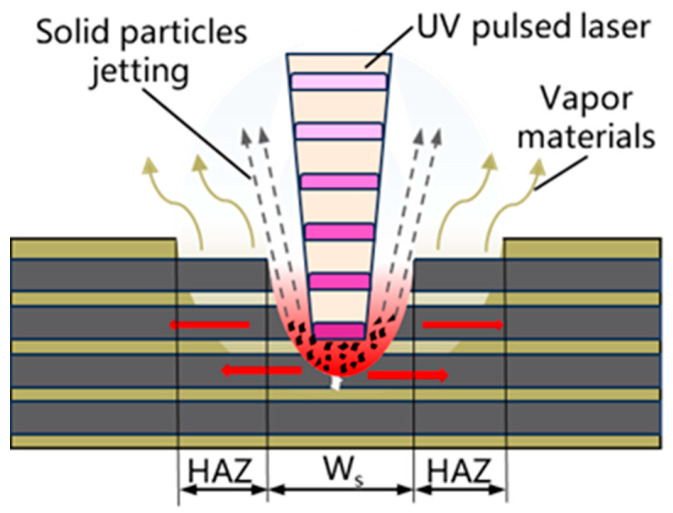
Schematic illustration of the interaction mechanism between the nanosecond UV laser and CF/PEEK.

Based on the orthogonal experiment, the microscopic morphology images, solid and gas products, combined with the basic principle and thermal effect of laser on materials, the mechanism of UV nanosecond laser cutting CF/PEEK was systematically analyzed. As shown in Figure 12, when the UV laser is irradiated to the surface of the material, the matrix absorbs energy first, undergoes photothermal decomposition, leaves the surface of the carbon fiber, and exposes the carbon fiber wire; since the evaporation temperature of the carbon fiber is much higher than the matrix, the exposed carbon fiber continuously absorbs energy. During this period, the carbon fiber conducts heat along the fiber direction, and the heat rapidly diffuses along the fiber direction and indirectly heats the PEEK, so that the matrix is vaporized and decomposed. Moreover, due to the concentration of laser energy, the local temperature of the fiber rises rapidly, so that some carbon fibers break and splash due to thermal stress, and some fibers continue to absorb energy. When the gasification temperature is reached, it decomposes and escapes. Moreover, the F_peak_ consistently remains on the order of 0.2 GW/cm^2^, which is below the typical threshold for significant plasma shielding induced by UV nanosecond lasers in air (generally > 1 GW/cm^2^) [32]. This further confirms that the photothermal effect dominates within the parameter range of this study.

## 4. Conclusions

On-orbit cutting is a necessary part of CF/PEEK truss on-orbit manufacturing. UV nanosecond laser cutting is suitable for CF/PEEK truss cutting due to its advantages of non-contact, high cutting accuracy, simple system composition, and strong material adaptability. In this paper, the cutting process of CF/PEEK was studied by ultraviolet nanosecond laser. Firstly, the quality evaluation system based on WS and HAZ is defined. Then, the optimal process parameters are obtained by orthogonal experiment and the influence of process parameters on cutting quality is analyzed. Finally, the ablation mechanism and ablation by-products are analyzed. The conclusions are as follows: The W_S_ and HAZ increase with the increase in LRR and LCT and the decrease in LCS. The influence of the LRR is greater than the LCS, and the influence of LCT is the smallest. Reducing the LRR and LCT, increasing the LCS can help to obtain better cutting quality, but it will affect the material removal rate. When the process parameters are 3 (20 kHz)—3 (50 mm/s)—3 (16), it can be completely cut off and achieve the optimal cutting quality (W_S_ = 41.69 ± 3.54 μm, HAZ = 87.27 ± 7.30 μm). During the processing, the carbon fiber decomposes and escapes due to the photothermal effect, and some fibers splash after breaking due to the thermal stress. The substrate is directly heated by the photothermal effect and indirectly heated by fiber conduction heat to form small molecule gas. Carbon fiber dust compromises equipment functionality; toxic gaseous by-products require stringent respiratory/eye protection during operations. The patterns revealed and the key data obtained in this study provide an important theoretical foundation and data support for subsequent systematic space environment simulation experiments.

## Figures and Tables

**Table 1:** Properties of CF/PEEK.

Parameters	Value
Material properties	CF/PEEK
Fiber model	T700
Fiber content	42 vol%
Thickness	0.15–0.25 mm

**Table 2:** Related parameters of nanosecond UV laser.

Parameters	Symbol	Value	Units
Wavelength	λ	355	nm
Average power	Pa	10	W
Pulse duration	τ	50	ns
Pulse frequency	F	10–300	kHz
Focused spot diameter	d_0_	37	μm
Focal length	f	167	mm
Beam quality	M^2^	1.2	

**Table 3:** Calculated values for typical parameters.

LRR (kHz)	*P_avg_* (W)	*E_p_* (mJ)	*F* (J/cm^2^)	*F_peak_* (GW/cm^2^)
10	1.18	0.118	10.98	0.220
20	2.20	0.110	10.23	0.205
30	3.16	0.105	9.80	0.196

**Table 4:** Factors and levels for the L25^(53)^ orthogonal experiment.

	Unit	Factor Levels				
		1	2	3	4	5
LRR	kHz	10	15	20	25	30
LCS	mm/s	10	30	50	70	90
LCT	—	8	12	16	20	24

**Table 5:** Results obtained from the L_25_(5^3^) orthogonal experimental design.

No.	LRR (kHz)	LCS (mm/s)	LCT	TS	HAZ (μm)	W_s_ (μm)
1	10	10	8	YES	88.50	27.20
2	10	30	16	NO	56.99	26.34
3	10	50	24	NO	61.31	27.11
4	10	70	12	NO	69.90	26.09
5	10	90	20	NO	59.84	23.04
6	15	10	24	YES	170.51	40.01
7	15	30	12	NO	111.44	44.19
8	15	50	20	NO	64.15	30.48
9	15	70	8	NO	60.86	22.12
10	15	90	16	NO	72.08	25.40
11	20	10	20	YES	158.86	47.72
12	20	30	8	YES	151.82	47.05
13	20	50	16	YES	87.27	41.69
14	20	70	24	YES	94.93	41.91
15	20	90	12	NO	53.80	24.65
16	25	10	16	YES	210.05	46.37
17	25	30	24	YES	163.15	48.38
18	25	50	12	YES	134.76	44.59
19	25	70	20	YES	92.96	41.87
20	25	90	8	NO	71.54	26.37
21	30	10	12	YES	168.62	50.19
22	30	30	20	YES	190.82	46.18
23	30	50	8	NO	176.38	47.02
24	30	70	16	YES	133.80	61.58
25	30	90	24	YES	130.47	54.29

**Table 6:** ANOVA test for HAZ.

Factor	SS	DOF	MS	F	*p*
LRR	101,373.26	4	25,343.31	588.99	<0.001
LCS	89,045.94	4	22,261.48	517.36	<0.001
LCT	3220.36	4	805.09	18.71	<0.001
LRR*LCS	37,216.33	12	3101.36	72.08	<0.001
SSe2	3227.15	75	43.03		

**Table 7:** ANOVA test for W_S_.

Factor	SS	DOF	MS	F	*p*
LRR	8712.02	4	2178.01	153.36	<0.001
LCS	2249.61	4	562.40	39.60	<0.001
LCT	782.96	4	195.74	13.78	<0.001
LRR*LCS	1476.88	12	123.07	8.67	<0.001
SSe2	1065.18	75	14.20		

**Table 8:** Composition table of the pyrolytic overflow gases.

Peak	RT	Area%	Name	Molecular Formula	Two-Dimensional Chemical	CAS
1	1.471	1.333	Carbon dioxide	CO_2_	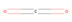	000124-38-9
2	1.762	0.974	1,3-Cyclopentadiene	C_5_H_6_	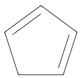	000542-92-7
3	2.335	7.890	Benzene	C_6_H_6_	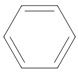	000071-43-2
4	6.851	42.506	Phenol	C_6_H_6_O	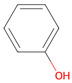	000108-95-2
5	10.135	1.367	Naphthalene	C_10_H_8_	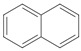	000091-20-3
6	12.923	1.611	Biphenyl	C_12_H_10_	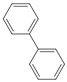	000092-52-4
7	13.198	3.218	Diphenyl ether	C_12_H_10_O	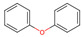	000101-84-8
8	14.700	6.647	Dibenzofuran	C_12_H_8_O	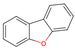	000132-64-9
9.	15.570	1.494	Isophorone diisocyanate	C_12_H_18_N_2_O_2_	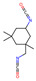	004098-71-9
10	16.040	1.265	Benzophenone	C_13_H_10_O	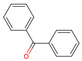	000119-61-9
11	21.712	1.700	p-Diphenoxybenzene	C_18_H_14_O_2_	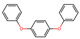	003061-36-7
12	22.944	1.476	2,3-anthracenedicarbonitrile, 1,4-dihydroxy-	C_16_H_8_N_2_O_2_	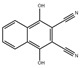	1000396-79-4
13	23.964	2.296	4-Hydrazono-5-hydroxyimino-4,5,6,7-tetrahydrobenzofuroxane	C_6_H_7_N_5_O_3_	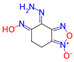	303194-86-7

## Data Availability

The original contributions presented in this study are included in the article. Further inquiries can be directed to the corresponding author.

## Abbreviations

The following abbreviations are used in this manuscript:

ANOVA	Analysis of variance
CF/PEEK	Carbon fiber reinforced polyetheretherketone composites
E_p_	The single-pulse energy
F	The single-pulse fluence
F_peak_	The peak fluence
HAZ	Heat-affected zone
LRR	Laser repetition rate
LCS	Laser cutting speed
LCT	Laser scanning times
P_avg_	The average power
Py-GC/MS	Pyrolysis–gas chromatography–mass spectrometry
R	The range
SEM	Scanning electron microscope
TG-IR	Thermogravimetric–infrared spectroscopy
UV	Ultraviolet
Ws	Width of slit
